# Motivational Drivers for Teachers as Informal Health Educators to Initiate In-Class Discussions With Adolescents About Smoking: Moderated Mediation Study Using Attribution Theory

**DOI:** 10.2196/81959

**Published:** 2026-04-17

**Authors:** Anna Joy Russ, Anna Bullo, Peter J Schulz

**Affiliations:** 1 Faculty of Communication, Culture and Society Università della Svizzera Italiana Lugano Switzerland; 2 Wee Kim Wee School of Communication and Information Nanyang Technological University Singapore Singapore; 3 Department of Communication & Media Ewha Womans University Seoul Republic of Korea

**Keywords:** attribution theory, in-class health communication, informal caregiver, smoking prevention, teachers

## Abstract

**Background:**

Teachers have the potential to be influential figures in school-based health promotion as informal caregivers; yet little is known about what motivates them to initiate preventive conversations with students. Attribution theory offers a useful framework to explore how perceptions of responsibility shape communicative behavior, but it has rarely been applied in the context of teacher-student interactions around health risks such as smoking.

**Objective:**

This study applies the attribution theory to explore the motivational drivers that lead teachers to initiate discussions with adolescents about smoking.

**Methods:**

Data were collected from 101 middle schools in the Canton of Ticino, Switzerland, as part of a larger longitudinal study. The analysis focuses on 67 teachers who participated in the first wave. Responsibility attribution, concern, and previous classroom sanctions were examined in association with teachers’ communication.

**Results:**

Results from a moderated mediation model showed that teachers who attributed greater responsibility to the school (internal attribution) reported higher levels of concern (*β*=–0.41; *P*=.002) and engaged in more frequent in-class discussions on smoking (*β*=–0.26; *P*=.02). Although concern alone was not directly related to communication (*β*=–0.14; *P*=.22), its effect was significantly moderated by contextual sanctions (*β*=1.01; *P*<.001).

**Conclusions:**

These findings highlight the motivational and contextual factors that shape teachers’ communication with students on smoking behavior. By applying attribution theory in the novel context of health communication, this research contributes to understanding how perceived responsibility influences preventive communication in schools.

## Introduction

### Overview

Imagine being a teacher (if you are not already one) faced with the challenge of addressing the topic of health prevention with your students. Now imagine feeling directly responsible for these young individuals, knowing that their future may, in part, depend on what you teach them, and becoming aware of the weight your words might carry in shaping their behavior. It is reasonable to assume that such awareness, this sense of responsibility, would significantly influence what you say in the classroom to prevent adverse behaviors, especially if your class is known for behavioral issues or has previously faced disciplinary actions. Conversely, suppose that while looking at these 20 or so faces, you are convinced that students’ health is entirely the responsibility of their parents. You are convinced that your role does not extend to teaching healthy behaviors, and you therefore feel no personal involvement in their long-term health. In such a case, you would likely be less inclined to engage with them in conversations aimed at preventing risky behaviors.

This is precisely the central focus of the research presented in this study: it concerns the motivational foundations of in-class teachers’ communication on smoking and applies attribution theory, originally developed by Heider [1] within psychology, to the novel and previously unexplored domain of teachers’ health communication with students in educational settings.

Research on the impact of communication on adolescent smoking has traditionally emphasized the influence of parents and peers [2]. Teachers, on the other hand, spend a similarly significant amount of time with adolescents but have received comparatively limited attention [3-5]. The school environment has been broadly considered in previous research [6,7]. What has been overlooked is the specific role of teachers in deterring adverse behaviors, with some early exceptions [8].

To address this gap, this research draws on the longstanding psychological framework of attribution theory to explore the mechanisms that may prompt teachers to initiate conversations about smoking. Although attribution theory has been successfully applied to explain pupil motivational drive or achievement evaluation in educational contexts [9-11], its use in communication research, particularly health communication, remains limited. Notably, in their contribution to the book *Communication and Learning*, Waldeck and Labelle [11] propose future research directions that include “extending its use to examine the relationship of instructor attribution of student behavior of corresponding communication behaviors both in and outside the classroom environment” (page 70). Although more than a decade has passed, this research cue has not yet been pursued. This study directly responds to this call by demonstrating the value of applying attribution theory to teachers’ interactions with students in the context of smoking prevention, contributing to a sparsely developed body of literature on teachers as active agents in health-related communication.

The following sections provide a detailed exploration of the literature supporting this inquiry, including teachers’ role in adolescents’ health, the context of adolescent smoking, and, finally, past work that has applied attribution theory.

### Teachers’ Role

Schools have long been the focus of numerous intervention programs aimed at reducing adolescent smoking [12,13]; yet the specific role of the adults who share this environment with students—namely, teachers—remains understudied [2,8]. Anecdotal evidence highlights the potential influence teachers can exert on students; many can recall a particular teacher whose words or actions left a lasting impression. This influence is unsurprising given that adolescents spend approximately 5 to 7 hours per day in close interaction with teachers during critical developmental years. Moreover, teachers can impact learning across cognitive, affective, and behavioral domains [14,15].

Teachers play a central role in adolescents’ lives, acting as a bridge between the adult world, represented by institutions and society, and the private, familial world of childhood [8]. Importantly, teachers are both accessible and cost-effective to involve in health-related initiatives, given that their professional role already encompasses a form of authority that is qualitatively distinct from that of parents [6]. Furthermore, their position grants them a dual role: as socializers guiding adolescents into adulthood and as communicators capable of voicing students’ needs to other adults. This dual function highlights their unique capacity to mediate between adulthood and childhood [8].

The teacher-student relationship has been shown to significantly affect a variety of health behaviors [16]. For example, Voisin et al [17] found that students reporting high levels of teacher connectedness were less likely to engage in adverse behaviors such as gang membership, risky sexual encounters, and substance use. Communication, specifically, has been identified as a key factor in enhancing the efficacy of intervention programs. Effective teacher communication has been defined as a set of communicative behaviors that improve learning [18]; this concept can be extended beyond academic topics to include health-related behaviors. In the study by Mesman et al [19], clear communication from teachers improved outcomes in interventions targeting alcohol use and physical activity. These findings suggest that a similar influence may extend to other health behaviors, such as smoking, although further studies will need to confirm this relationship.

Understanding the motivational processes that drive teachers to engage with students in conversation about health behavior is crucial, as it opens the door to developing strategies that support and enhance teacher-led communication as a form of school intervention. This is precisely why the theoretical framework adopted in this study is particularly well suited: to effectively increase teacher engagement in preventive communication, efforts must target the motivational grounds for such communication. Among these, perceived responsibility for adolescent health can be a promising factor. By increasing the perception of responsibility, it could be possible to foster more frequent and meaningful communication, ultimately leading to a higher impact of prevention on adolescent smoking.

### Adolescents’ Smoking

The urgency of addressing adolescent smoking stems not only from its well-documented health consequences, such as respiratory disorders, cardiovascular diseases, and cancer, as highlighted by the review by Arafa et al [20], but also from its negative effects on cognitive development. Tobacco use during adolescence has been shown to hinder the development of brain networks and cognitive functions. Nicotine products are, without a doubt, toxic to the neurodevelopment of young people [21-23].

Beyond its impact on individual health, the global tobacco industry contributes significantly to environmental degradation, including deforestation, air and water pollution, biodiversity loss, and greenhouse gas emissions [24-26]. As such, smoking represents a critical behavior to target, not only for personal health but also for environmental protection. Preventing smoking initiation during adolescence is especially critical, as early smoking onset significantly increases the likelihood of long-term tobacco consumption [27].

Recent data confirm that adolescent smoking remains a pressing global public health issue, with notable prevalence rates reported even in high-income countries with strict regulations. For example, the Health Behavior in School-Aged Children study indicates that a substantial percentage of youth experiment with or regularly use tobacco products, with nearly a quarter of adolescents aged 15 years reporting having ever smoked [28]. Adolescent smoking is strongly influenced by psychosocial factors such as peer norms, social identity, and stress—factors that make this period especially critical [29-32].

This underscores why the timing of intervention is crucial: adolescence is a developmental window during which risk-taking increases [33]. In this context, teachers are unique figures who can influence adolescent health trajectories. Their presence and authority place them in a powerful position to support early prevention efforts. Moreover, addressing adolescent smoking cannot be left solely to families or health care systems. Schools represent a critical institutional setting for early prevention, not only because they welcome a great variety of young people but also because they provide opportunities for sustained interaction between students and educators, which can foster trust and influence over time.

### Attribution Theory

This study applies attribution theory, first developed by Heider [1] and further elaborated by Weiner [34]. It has been successfully used for decades in motivational research, social psychology, and educational psychology [35], but has rarely been applied in the context of health communication. According to this theory, individuals ascribe causality, and subsequently responsibility, to events and behaviors in their everyday lives [36]. These attributions, often made unconsciously, help understand why people do what they do and what motivates them to engage or disengage in specific behaviors [37].

Attributions are shaped in accordance with preexisting beliefs and are characterized by three distinct dimensions: (1) locus of causality, (2) stability, and (3) controllability [9,35,37-40]. The locus of causality refers to whether the cause of an event is attributed to personal characteristics within one’s control (ie, internal attributions) or external factors beyond one’s control (ie, external attributions) [38-41]. The stability dimension, as the name suggests, refers to the perceived consistency of the cause over time, whether it is a fixed trait or a fluctuating condition [38,39]. Lastly, the controllability dimension concerns whether the cause is perceived as subject to personal control and is closely tied to perception of personal responsibility [38]. Although this dimension was not part of the original formulation—as it was added by Fiske [42]—it has since been included as a “proximal critical cognitive consequence” of causal attribution [38,43]. This last dimension is the focal point of the research presented here.

In practice, individuals evaluating a situation unconsciously assess the cause of an event or behavior, including how responsible they feel in that situation [44]. This attribution directly shapes their behavioral response, as each evaluation elicits distinct psychological and behavioral consequences, such as help giving or help seeking [9,44].

**Figure 1:**
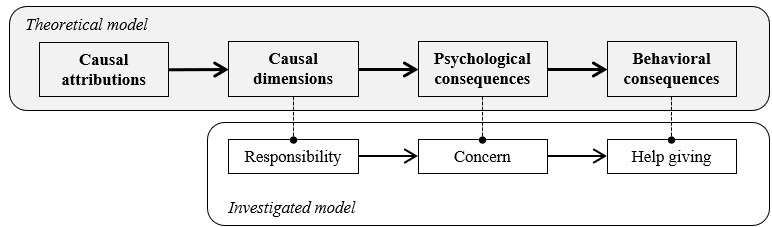
Representation of the attributional model tested.

The model tested in this paper is depicted in Figure 1 and shows the linear framework outlining the pathway from causal attribution to causal dimensions, then to psychological consequences, and lastly to behavioral consequences. The linear framework outlines the pathway from causal attribution to causal dimensions, then to psychological consequences, and lastly to behavioral consequences. This study focuses on the final 3 components of the model to examine their relevance within the teacher-student communication context. Specifically, the causal dimension triggered by the attribution is the feeling of personal responsibility; the psychological consequence is the rise of the social emotion of concern; and the behavioral consequence is help giving, through the form of communication [9].

Exploring each component in more detail, the attribution of responsibility in educational contexts has been the subject of several studies, with both students and teachers as attributers [45-47]. In one section of the systematic review conducted by Wang and Hall [38] focused on teachers’ attributions, the authors present 9 papers that specifically examine teachers’ perceptions of responsibility. However, the studies presented are exclusively on responsibility in relation to academic performance, without addressing either health-related themes or the communicative behaviors between teachers and students.

Regarding the psychological consequences of the causal attribution, concern has been selected as a social emotion, consistent with the definition given by Burnett et al [48] as an emotion that involves mentalizing about other people. Concern arises in social contexts in which individuals affect one another’s emotional state [49]. In the context of this study, the emergence of teacher concern for students is expected to be influenced by whether teachers feel responsible for their students’ health behavior.

Lastly, help giving has been operationalized as communication: the first step in supporting students within the context of smoking prevention is the act of speaking to them about the issue and drawing their attention to its risks. Whether through advice, emotional support, or information sharing, communication can fulfill the function of helping others, particularly in contexts in which direct intervention is limited. This has been demonstrated in studies on doctor-patient communication and online support groups [50-52]. In the case of smoking prevention, the most accessible and powerful tool teachers possess—aside from disciplinary measures—is their ability to educate adolescents about the harmful effects of tobacco use. Teachers’ communication skills have been consistently associated with more effective learning outcomes [53] and the success of intervention programs [19,54]. Moreover, training in communication is frequently identified as a strategy for improving teacher efficacy [55]. Together, these findings underscore the central role of communication as a vehicle for preventive action in school settings.

Building on the theoretical framework presented thus far, we can summarize the core logic: hypothetically, teachers who feel personally responsible for preventing smoking among their students should experience greater concern than those who place responsibility externally. This heightened concern should in turn increase their motivation to protect students through communicative engagement in the classroom. In this way, communication could become the behavioral outcome of an attribution-based emotional and cognitive process—bridging the sense of responsibility and the intention to help students. This framework highlights the relevance of attribution theory in explaining the motivational base for health-related communication.

### Contributions of This Study

Based on the literature review, several gaps emerge that this study seeks to address. The first concerns the attribution of responsibility by teachers: no past research, to the best of the authors’ knowledge, has uncovered who is deemed responsible in the eyes of teachers for smoking prevention. Furthermore, in the context of tobacco consumption, it is not known how the attribution of responsibility affects communicative behavior: the motivational drivers behind teachers’ decisions to engage in conversations with students about smoking remain underexplored. While existing research provides some evidence of teachers’ influence on adolescent health behaviors, little is known about their personal motivators or decision-making processes in initiating such communication. To address this, the study tests the linear model proposed by Graham [9], grounded in Weiner’s [39] attribution theory, focusing on concern as a social emotion that mediates the relationship between attribution of responsibility and communication.

In addition to this theoretical contribution, the study introduces a novel contextual variable not previously discussed in the literature review: the smoking behavior of students. This variable has been operationalized in this study as the presence or absence of prior sanctions for smoking, considering that this information implicitly includes the teachers’ perception of classroom smoking behavior. This element is hypothesized to moderate the relationship between teachers’ concern and their likelihood of communicating about smoking. Specifically, the expectation is that highly concerned teachers will be less likely to initiate discussions in classrooms in which no sanctions have previously been applied compared with those teaching in classes with a history of disciplinary action related to smoking. This reflects the idea that contextual cues, such as prior disciplinary actions, may shape whether emotional concern is translated into communicative behavior.

These considerations lead to the development of the study’s central research question and hypotheses, summarized in Table S1 in Multimedia Appendix 1. They aim to empirically test both the motivational and contextual factors driving teacher-student communication on smoking prevention.

### Research Question and Hypotheses

The study was guided by the following research question (RQ) and hypotheses (HP):

- RQ1: To whom do teachers attribute responsibility for preventing students from smoking?
- HP1: Attributing responsibility to the school context (ie, internal locus of responsibility) is associated with engaging in more discussion on smoking with students compared with attributing responsibility to parents (ie, external attribution of responsibility).
- HP2: Concern about future problems resulting from smoking (M), as a psychological consequence, will mediate the link between responsibility attribution (X) and in-class communication (Y).
- HP3: The presence or absence of sanctions applied to the class will moderate the relationship between concern and communication. Specifically, higher concern is expected to be associated with more frequent communication when students have previously received sanctions for smoking.

## Methods

### Ethical Considerations

The study was conducted according to the guidelines of the Declaration of Helsinki. The questionnaire and methodology for this study were approved by the Cantonal Bureau for Education in the Canton of Ticino, Switzerland (September 2017). No exemptions from ethics review were applicable. Written informed consent was obtained from participating teachers at the beginning of data collection; participants were informed that their participation was voluntary and that they could withdraw at any time without explanation. To protect their privacy, all data were anonymized using alphanumeric codes. No financial or other compensation was provided.

### Procedure

This research is part of a larger longitudinal study involving teachers, parents, and adolescents from a representative sample of 101 middle schools in the Canton of Ticino, Switzerland. The Canton of Ticino is located in the southern part of Switzerland, and the official language is Italian. As of 2024, the canton accounts for 4% of the total Swiss population (354,023 permanent residents), and the proportion of young people aged 15 years or younger has been steadily decreasing, reaching 12.6% of the total population in 2023 [56].

The study aimed to investigate the evolution of various health behaviors from the perspective of adolescents, their parents, and their teachers.

In September 2017, marking the beginning of data collection, a random sample comprising 66% of all first-year public school classes from the 5 areas of the canton was selected. The school sample was randomly selected to ensure representation of the broader school population. Private schools were also invited to participate; 1 of 3 agreed to participate, and 1 class was randomly selected. The final sample consisted of 101 classes from 36 schools (35 public and 1 private). Each class designated a reference teacher who could voluntarily participate in the study. The data analyzed in this study refer to the 67 teachers who voluntarily participated in the first wave of data collection, conducted at the end of the 2018 school year. The response rate was 66.3%, which is relatively high given that the questionnaire was administered at the end of the academic year, when teachers are usually engaged in closing activities and planning for the summer recess.

Paper questionnaires were distributed to all participating schools for completion by adolescents, parents, and teachers. Reference teachers were asked to complete the survey at the end of school board meetings, ensuring they had an up-to-date understanding of the classroom context. Once completed, they were asked to return the questionnaires to the university using a preaddressed, stamped envelope.

### Participants

Of the 67 teachers who participated, 60% (40/67) identified as female, 30% (20/67) as male, and 10% (7/67) did not disclose their gender. In 88% (56/67) of the cases the role of reference teacher was assumed by the class teacher, a key figure in educational system of Ticino, responsible for mediating between the school and the families, as well as evaluating students’ performance and workload [57]. The remaining respondents (11/67, 12%) identified themselves as members of management or as subject-specific lecturers. Participating teachers taught their class for a minimum of 2 hours and a maximum of 10 hours per week (mean 4.98, SD 2.10 hours), and more than three-quarters (41/67, 77%) had at least 5 years of teaching experience in middle school. Overall, teachers reported an average of 11.8 (SD 8.41) years of teaching experience and a mean employment rate of 81.3% (SD 22.90%).

### Materials

#### Overview

The questionnaire used in the longitudinal study included both validated scales and ad hoc items developed specifically for this data collection. The following section describes the variables included in the model presented in this study.

#### Responsibility Attribution

Attribution of responsibility was measured using a set of 8 items covering both health-related and educational behaviors. For this study, only the item related to smoking was analyzed. Participants responded to the general prompt, “In your opinion, which of the following educational goals are the responsibility of the school, and which are the responsibility of the parents?” with 1 of the specific goals being: “Ensuring that children do not smoke.” Responses were rated on a 7-point Likert scale, where 1 indicated full responsibility attributed to the school, 4 indicated shared responsibility, and 7 indicated full responsibility attributed to parents. Responses attributing responsibility to the school were considered an internal attribution, as teachers are part of the school context and their answers reflect both their own behavior and their awareness of institutional norms and practices. Conversely, attributing full responsibility to parents was regarded as an external attribution.

#### Concern

Teachers’ concern was assessed with the following item: “How concerned are you that the following behaviors may lead middle school students to have problems in their future lives?” A list of 8 adverse behaviors was provided, including “cigarette consumption.” Responses were recorded on a 5-point Likert scale (1=not at all and 5=very).

#### Sanctions

Teachers were asked to consider the past academic year and indicate “on how many occasions students in the class [insert class evaluated] were appropriately sanctioned if they performed any of the following behaviors.” Sanctions could have been issued by the teacher himself or by a colleague. For this study, only the responses for smoking cigarettes were used. The response option was a 6-point Likert scale (0=this type of incident never occurred and 6=always). As the item, in its original formulation, contained two types of information—(1) the presence or absence of sanction and (2) the appropriateness of the sanctions—the researchers dichotomized the variable to isolate the first type of information. In this recoding, a response of 0 was retained as reference category to indicate that no sanctions were applied, while all other response options (1 through 6) were recoded to 1, signifying that a sanction was given to the class.

#### Communication on Smoking

The amount of communication in the classroom regarding health issues was measured with the following item: “During this school year, how often have you or other teachers discussed the following issues with the class [insert class evaluated]?” A list of 4 topics was presented, including tobacco use. Teachers could respond on a 4-point scale (1=never and 4=often).

#### Hours of Teaching (Covariate)

To control for time spent with the students, teachers were asked to indicate the number of hours they taught each week in the classroom being evaluated. This variable was treated as continuous.

### Data Analysis Plan

The required sample size was determined using G*Power (version 3.1.9.7) [58]. A minimum of 51 participants would be needed with 95% power at α=.05 (2-tailed). The final sample for the analysis included n=59, exceeding the requirement.

Using SPSS Statistics (version 29; IBM Corp), descriptive statistics were applied to analyze the variables under consideration. Means, frequencies, percentages, and SDs were used to describe the data, as well as to answer the first research question (RQ1). Pearson correlations were conducted to assess the relationship between the variables, and a 2-tailed *t* test was performed to examine differences in the continuous variables by the dichotomous variable of sanctions.

The hypothesized regression paths (HP1, HP2, and HP3) were tested using Model 14, a moderated mediation model, of Hayes PROCESS macro in IBM SPSS Statistics (version 29), with 10,000 bootstrap samples [59]. The analysis consisted of a regression test on the effect of responsibility attribution on communication frequency, as well as a test on the role of concern in mediating this relationship at the 2 levels of reported sanctions (ie, no sanctions and presence of sanctions). The mediator (concern) was mean centered prior to analysis. The variable “hours of teaching spent in the classroom” was used as a control variable. The model was tested with the addition of several teacher-level variables (ie, gender and years of experience) but yielded nonsignificant results and deteriorated overall model fit. Therefore, the more parsimonious model was retained. The graphical representation of moderation was performed in R (version 4.5.0; R Foundation for Statistical Computing) using the package *interactions* [60].

## Results

### Descriptive Results

Descriptive statistics are reported in Table 1. Following the recommendations of VanderWeele [61], descriptive statistics stratified by sanction level are reported to facilitate interpretation of interaction effects. Correlations and additional information are provided in Table S2 in Multimedia Appendix 1. Information on the variable responsibility attribution will be discussed in the next section, as it directly relates to RQ1. The variable concern had a mean of 2.97, indicating a moderate level of concern for future problems due to smoking. The distribution appears approximately normal, with minimal skewness (0.17) and kurtosis (–0.70). A similar pattern was observed for in-class communication on smoking, for which the mean score was 2.09, reflecting a low to moderate frequency of communication, and the variable appears normally distributed (skewness=0.41 and kurtosis=–0.92). Sanctions were given in 20% (14/67) of cases.

**Table 1:** Descriptive statistics for continuous variables, stratified by sanctions.

Variable	Frequency, n	Mean (SD)	Range	Sanctions, mean (SD)	
				Yes (n=14)	No (n=53)
Responsibility attribution	65	4.77 (1.07)	3-7	4.43 (0.85)	4.86 (1.12)
Concern	67	2.97 (1.18)	1-5	3.43 (0.94)	2.85 (1.22)
Communication	67	2.09 (0.98)	1-4	2.71 (1.32)	1.92 (0.81)
Hours of teaching (covariate)	61	4.98 (2.10)	0-10	5.46 (2.18)	5.07 (1.86)

Regarding correlations, communication frequency was significantly associated with all variables: it was positively correlated with concern (*r*=0.25; *P*<.05) and negatively correlated with responsibility attribution (*r*=–0.26; *P*<.05). The independent-samples 2-tailed *t* test revealed a significant difference in communication scores between sanction condition (mean 2.71, SD 1.33) and the no-sanction condition (mean 1.92, SD 0.80); t_65_=–2.82; and *P*=.003. Concern and responsibility attribution were not differently distributed in the 2 conditions of sanctions. In other words, higher levels of communication were associated with greater concern, a more internal attribution of responsibility, and the presence of sanctions.

### Main Results

Most teachers in the sample (36/67, 54%) attributed a shared responsibility for smoking prevention, and the descriptive statistics offer further insight into this pattern (RQ1). The mean of 4.77 (SD 1.07) suggests that, on average, teachers tend to lean toward attributing more responsibility to parents than to the school, as higher scores reflect external attribution. The distribution is slightly skewed, indicating more teachers score toward the higher end of the scale (Table 1). Notably, no participant attributed full responsibility to the school alone, while 10% (7/67) of participants stated that smoking prevention would solely involve parents.

**Figure 2:**
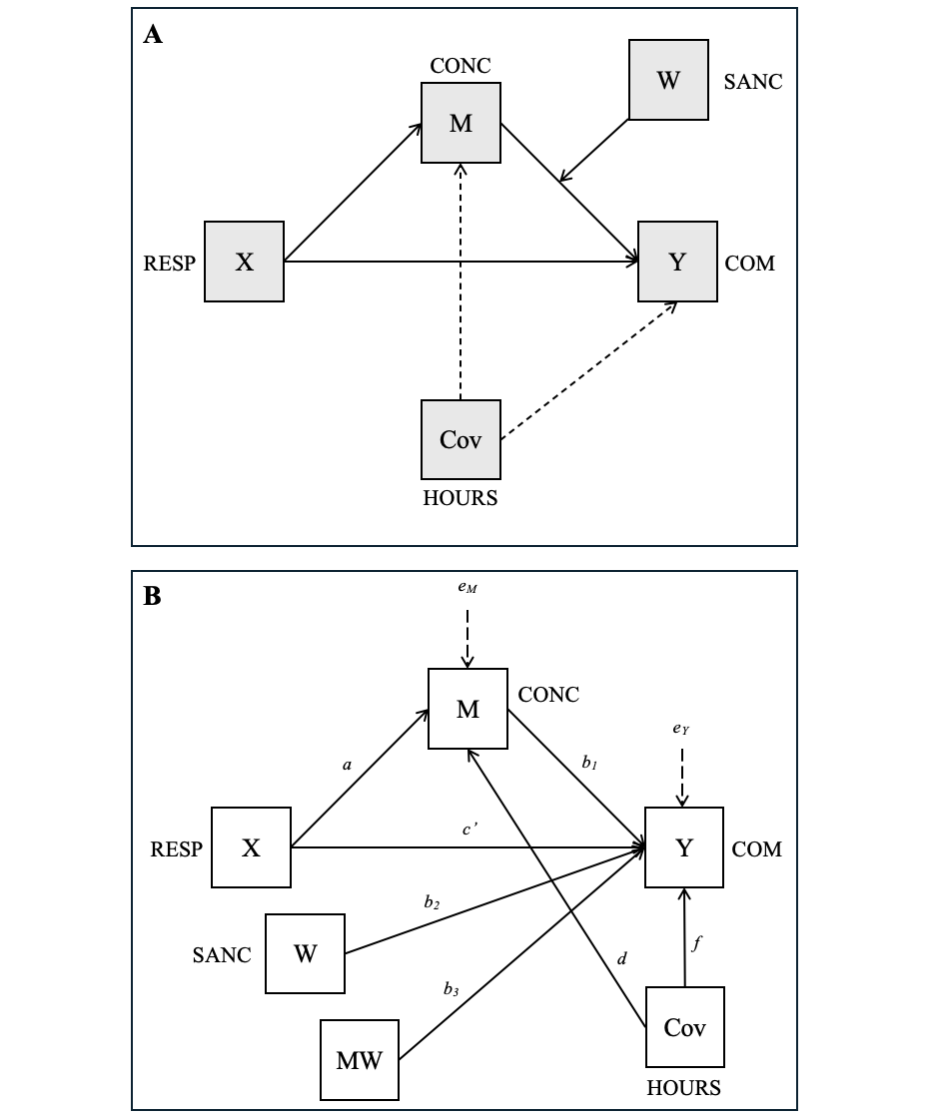
The (A) conceptual and (B) statistical representation of the moderated mediation model. COM: communication; CONC: concern; M × W: interaction effect; M: mediator; RESP: responsibility attribution; SANC: sanctions. W: moderator; X: independent variable; Y: dependent variable.

Using Model 14 of the PROCESS macro [59], it was possible to test the 3 hypotheses. The conceptual and statistical diagrams of the tested model are shown in Figure 2, while the results of the analysis are presented in Table 2. In terms of model fit, a moderate amount of variance was explained, particularly for the outcome variable communication (39%). Controlling for weekly hours of teaching did not change the direction, strength, or significance of the main effects. Both the mediator (M; concern) and the dependent variable (Y; communication) were significantly predicted by the model, as indicated by the respective *P* values.

**Table 2:** Model coefficients for the moderated mediation model.

Antecedent	Consequent									
	Path	M^a^ (CONC^b^)^c^				Path		Y^d^ (COM^e^)^f^		
		Coefficient	SE	*P* value			Coefficient		SE	*P* value
X^g^ (RESP^h^)	a	–0.41	0.13	.002	c'		–0.26		0.11	.02
M (CONC)	—^i^	—	—	—	b_1_		–0.14		0.12	.22
W^j^ (SANC^k^)	—	—	—	—	b_2_		–2.56		0.95	.01
M × W^l^	—	—	—	—	b_3_		1.01		0.27	<.001
Constant	i_M_	4.26	0.74	<.001	i_Y_		3.17		0.76	.001
Covariate (hours)	D	0.11	0.07	.13	f		0.07		0.06	.25

^a^M: mediator.

^b^CONC: concern.

^c^*R*^2^=0.19; *F*_2,56_=6.71; *P*=.002.

^d^Y: dependent variable.

^e^COM: communication.

^f^*R*^2^=0.39; *F*_5,53_=6.83; *P*=.001.

^g^X: independent variable.

^h^RESP: responsibility attribution.

^i^Not applicable.

^j^W: moderator.

^k^SANC: sanctions.

^l^M × W: interaction effect.

Before delving into hypotheses testing, it is important to note that, because the data are cross-sectional, the directionality of the observed relationships cannot be established. HP1 was supported: the path from responsibility attribution (X) to amount of communication (Y) showed a significant negative direct effect (*β*=–0.26; *t* value=–2.35, *df*_1_=5, *df*_2_=53; *P*=.02; 95% CI –0.47 to –0.04), even when controlling for concern and interaction effects. The outcome model accounted for a substantial proportion of variance in communication (*R*²=0.39; *F*_5,53_=6.83; *P*=.001). In practical terms, teachers who reported higher scores on responsibility attribution (ie, with an external focus, attributing more responsibility to parents) were also associated with lower rates of in-class discussions on smoking.

The mediation effect (HP2) was not confirmed in the main effect but supported under contextual moderation. As a matter of fact, the effect of responsibility attribution (X) on concern (M) was significant and negative (*β*=–0.41; *t* value=–3.22, *df*_1_=2, *df*_2_=56; *P*=.002; 95% CI –0.66 to –0.15), indicating that external attribution was associated with lower teacher concern. However, the main effect of concern (M) on in-class communication (Y) was not significant (*β*=–0.14; *t* value=–1.23, *df*_1_=5, *df*_2_=53; *P*=.22; 95% CI –0.38 to 0.09). In other words, unloading the feeling of responsibility to parents was linked to teachers being worried about the future of their students, but this was not directly associated with a decreased communication effort. Concern alone was not significantly related to communication.

**Figure 3:**
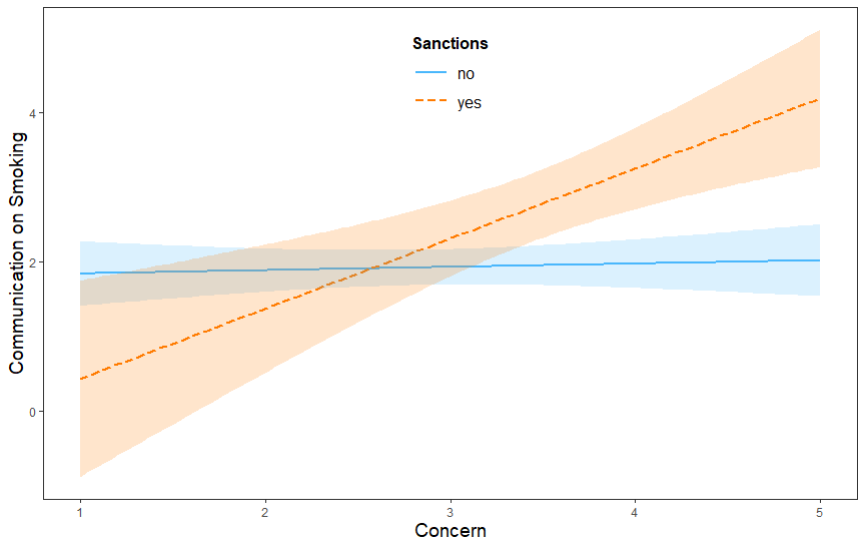
Graphical representation of the moderation effect on communication.

In contrast, HP3 was confirmed: the effect of concern (M) on communication (Y) was indeed moderated by the contextual factor of sanctions (W). The interaction was statistically significant (*β*=1.01; *t* value=3.74, *df*_1_=5, *df*_2_=53; *P*<.001). When no sanctions were present, the indirect effect was not statistically significant (effect=0.06, 95% CI −0.02 to 0.16). In contrast, when sanctions were present, the indirect effect was significant and negative (effect=−0.35, 95% CI −0.64 to −0.07), indicating that external attribution reduced concern, which in turn reduced communication in sanctioned classrooms. This revealed how concerned teachers talked significantly more in classes where students had been punished for smoking in the previous academic year, compared with students in classes with no sanctions. Further information on the direct and indirect effects is available in Tables S3 and S4 in Multimedia Appendix 1. The indirect effect of responsibility attribution on communication through concern was significant only when past sanctions were present. When no sanctions were applied, the indirect effect was nonsignificant. Practically speaking, when the class had been sanctioned for smoking, the path connecting responsibility attribution to concern and finally to communication was significant (Figure 3).

## Discussion

### Overview

This study explored the motivational and contextual factors that influence teachers’ decisions to engage in classroom discussions about adolescent smoking, using attribution theory as its framework. Before delving into the discussion of the results, it is important to note that this is the first study to investigate the effect of responsibility attribution on communicative behavior. Given the absence of directly comparable studies in the existing literature, this section is primarily devoted to discussing the implications of the findings and proposing avenues for future research.

Findings related to the first research question, regarding whom teachers view as primarily tasked with deterring students from smoking, revealed that teachers generally attribute slightly more responsibility to parents than to the school. Considering parents are still seen by teachers as the reference figures for adolescents, this is not surprising [62]. The result is nevertheless promising and suggests that public efforts to involve teachers in health prevention are well aligned with teachers’ perceptions. While teachers do feel slightly less responsible than parents, the difference is minimal. This finding indicates that, in Switzerland, the role of health educator is appropriately positioned in the hands of teachers: they are expected to lead projects on health education and promotion, and several initiatives directly involve them in targeting adolescent populations [63]. Their role in prevention is therefore participatory and includes, but is not limited to, enforcing no-smoking policies in the school perimeter and applying sanctions when rules are broken [64]. The first key finding somewhat reflects this institutional commitment of being involved in children’s health prevention. The teachers’ feeling of responsibility is expected to vary across health topics, depending on how these issues are addressed in school programs. For example, topics such as sexual or mental health have been more explicitly and widely integrated into curricula through structured programs [65]. Testing their attributions and evaluating their differences when tackling health behaviors is therefore the first main further direction of research to pursue.

The results support the first hypothesis: teachers who attributed greater responsibility to the school (internal attribution) were more likely to communicate with students about smoking. This key finding has both theoretical and practical implications. Theoretically, it establishes the successful use of attribution theory to explain teachers’ communicative behavior and supports the idea that perceived responsibility is a key driver of preventive communication [9]. Practically, it highlights the motivational force behind such communication. Considering the model accounts for 39% of the variance, responsibility attribution appears to be a meaningful factor to consider in preventive efforts. A next step would be to examine how parents attribute responsibility and compare their patterns to those of teachers. Questions remain open on situations in which parents’ and teachers’ perception diverge. Furthermore, exploring adolescents’ perspective could offer interesting insights on who they recognize as authoritative and trustworthy figures in health communication. Perceptions of authority and trustworthiness will most likely impact the way adolescents perceive and respond to preventive efforts. In the case of teachers, future research could explore whether increased teacher communication correlates with reduced smoking rates, and under which relational circumstances this effect is most pronounced.

The relation between attribution and feeling of concern, which is the first part of the mediation process shown in Figure 2A, was confirmed: internal attributions were indeed associated with heightened apprehension about students’ future well-being. This finding aligns with the idea that such emotion is a social one: it arises in response to others and is shaped by relational context. Experiencing this form of emotional engagement appears to be a response to perceived responsibility. This supports the first stage of Graham’s [9] model, and highlights the potential value of testing other social emotions in future applications, such as pity, guilt, pride, or embarrassment [49,66]. The second hypothesis is, however, only partially confirmed, as concern alone was not significantly linked to communication, suggesting that further contextual clues, such as past sanctions, are needed.

Specifically, the moderated mediation is significant only in the “sanctioned” condition (Figure 2A): concern is associated with communication only when the class has a history of sanctions. Practically speaking, concerned teachers communicated more in classes where students had previously been sanctioned for smoking. This finding is not surprising, as it is reasonable to expect that prevention efforts are more likely to be directed toward students who have already exhibited problematic behavior. In contrast, in classes where smoking is not perceived as an issue, teachers may not feel the need to engage in preventive communication. These results show that classroom dynamics can shape how concern translates into action. The result prompts 2 considerations. First, teachers may not always be fully aware of their students’ health behaviors, potentially overlooking those who smoke but have not been caught. Second, reduced communication in such cases may leave students more vulnerable to a future smoking threat. As inoculation theory has demonstrated, consistent communication efforts help protect adolescents from smoking onset [67]. This opens the door to exploring other contextual variables that may shape this relationship, such as school norms or the nature of teacher-parent relationships.

### Implications

The research has 2 key implications, 1 related to theory and the other to practice. Theoretically, it successfully applies a psychological theory to the novel field of health communication, identifying attribution theory as a promising framework for studying the drivers of communicative behavior. Communication can be influenced by individuals’ responsibility attributions, and this approach is promising because it could be applied to various health-related communication involving multiple actors (eg, parents).

Practically, the findings underscore the vital role of teachers in smoking prevention, positioning them as key figures in adolescents’ lives. As previous research has consistently shown, adolescents are shaped by their surroundings—be it their neighborhoods, home environments, or schools—but particularly by the individuals who share these spaces with them [35,68,69]. By actively engaging teachers and empowering them to feel invested in their students’ futures, healthier behaviors in adolescents can be fostered.

### Limitations and Strengths

This study is not without limitations. First, the sample size of the participating teachers was relatively small, which may limit the statistical power of the findings. However, it is important to note that two-thirds of the schools in the Canton of Ticino were involved and randomly selected—a region that, to begin with, comprises a relatively small number of schools. Among these, the response rate was 66%, which is not low considering the questionnaire was administered at the end of the school year, when teachers were beginning their summer vacation. Second, the use of self-reported data introduces the possibility of social desirability or recall bias. Additionally, variables were measured with single-item scales, due to the constraints of the dataset, as the data analyzed in this study were originally collected for different research purposes. While the use of single-item measures can potentially reduce reliability, their application is not without precedent. Wanous et al [70], in their review of job satisfaction measures, concluded that although multi-item scales are generally preferable, single-item measures may be acceptable when justified by the research context. Furthermore, some items referred also to the school context and not to the individual teacher. We argue, however, that this approach is still valid. Teachers are embedded in the broader context of schools, and their responses likely reflect not only their own behavior but also their perception of institutional practices. Preventive initiatives are often implemented at the school level, and individual teachers’ perceptions may be shaped by what their colleagues are doing. The application of multilevel modeling techniques was considered but not implemented due to sample size constraints (ie, limited number of teachers per school). Future research should address this limitation by using larger samples that allow for such modeling.

A further limitation lies in the fact that the study was conducted within the specific sociocultural and educational context of the Canton of Ticino, which may limit the generalizability of the results to other regions or countries. Finally, due to the nature of the cross-sectional data, it is not possible to infer causality or directionality in the model tested.

Despite these limitations, the study offers several notable strengths. It presents a novel application of attribution theory to the field of communication, extending its use into educational settings to explain teacher-student interactions. This theoretical lens provides insights into the motivational drivers behind teachers’ communicative efforts regarding adolescent smoking. Moreover, the study draws on a representative sample of schools. Lastly, by focusing on teachers (an often overlooked yet influential population in prevention research), this work contributes to filling an important gap in the literature and opens new directions for theoretical exploration.

### Future Directions

This study opens several promising avenues for future research in applying this theoretical framework. First, future research may consider involving a more diverse sample of teachers to examine how variations in role, teaching experience, or educational context may influence perceived responsibility. A further direction would be to apply attribution of responsibility theory to other health-related topics, such as sexual health, to assess whether the strength or nature of the association differs across issues. To establish causal relationships, the current findings should be replicated using longitudinal data. Finally, the link between attribution of responsibility and communicative behavior could be explored in relation to other key actors, beginning with parents and extending it to the impact on adolescents’ behavior, to broaden the scope of this theoretical application.

### Conclusions

Let us once again imagine ourselves as teachers facing a classroom of students and talking to them about adverse health behaviors, precisely smoking. According to the results of this study, our level of perceived responsibility will influence our behavior, leading us to speak more frequently with our students when we feel directly responsible for their health. This will happen especially in cases in which concern is high and the class has previously shown issues related to tobacco use.

These findings are particularly significant because they show not only that acting on the perception of responsibility is a valid and effective way to increase classroom conversations about health topics, but also that this theoretical model has the potential to explain communicative behaviors across a range of contexts. A broader assumption of responsibility has the potential to increase communication on sensitive topics and thereby raise awareness on adverse behaviors in the minds of young adolescents.

## Data Availability

The datasets generated or analyzed during this study are available from the corresponding author on reasonable request.

## Appendix

Multimedia Appendix 1 Data on research question and hypotheses investigated and distribution statistics for continuous variables.

## Abbreviations

HP: hypotheses
RQ: research question
