# Supplementary material for: Motivational Drivers for Teachers as Informal Health Educators to Initiate In-Class Discussions With Adolescents About Smoking: Moderated Mediation Study Using Attribution Theory
Source: JMIR Pediatr Parent. 2026 Apr 17;9:e81959. doi: 10.2196/81959 (PMC13135160; doi:10.2196/81959)
Supplement: Multimedia Appendix 1 [file pediatrics_v9i1e81959_app1.docx]

**Appendix 1**

**Table S1.** Summary of research question and hypotheses investigated.

| Research question/Hypotheses | Graphical representation of path investigated | Final decision |
| --- | --- | --- |
| RQ1. Who do teachers attribute responsibility for deterring students from smoking? | 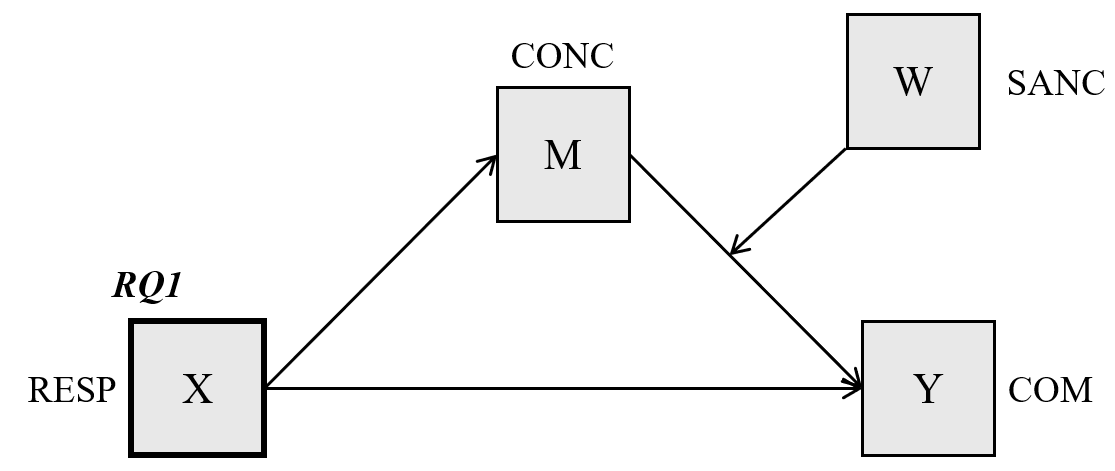 | Leaning toward more parental responsibility. |
| HP1. Teachers with an internal attribution of responsibility engage in more frequent in-class conversations about smoking. | 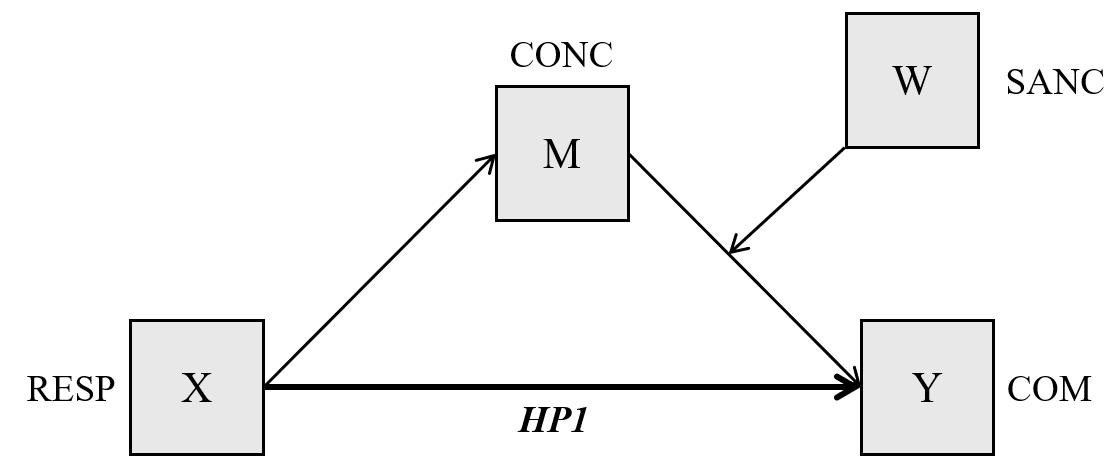 | Confirmed. |
| HP2. Concern mediates the relationship between attribution of responsibility and communication. | 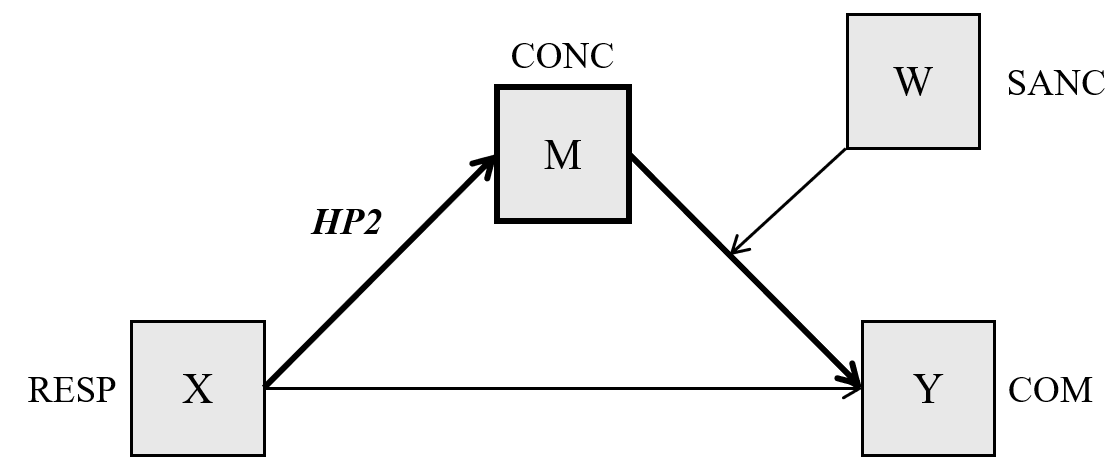 | Not confirmed in the main effect but supported under contextual moderation. |
| HP3. Sanctions will moderate the relationship between concern and communication. | 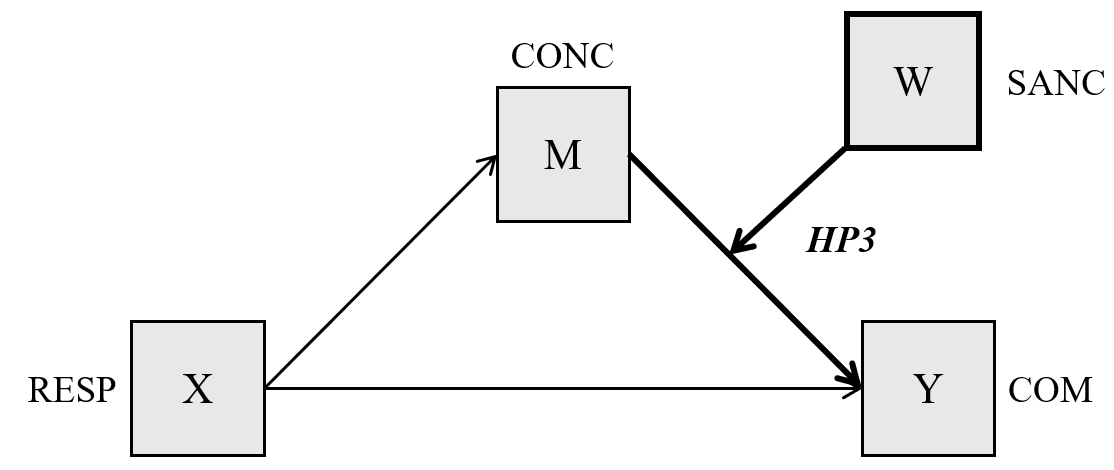 | Confirmed. |

Abbreviations: RESP = responsibility attribution; CONC = concern; SANC = sanctions; COM = communication.

**Table S2.** Correlations and distribution statistics for continuous variables

| Variable | Skewness (SE) | Kurtosis (SE) | 1. | 2. | 3. |
| --- | --- | --- | --- | --- | --- |
| 1. Responsibility attribution | .95  (.30) | –.33  (.59) | -- |  |  |
| 2. Concern | .17  (.29) | –.70  (.59) | –.39^a^ | -- |  |
| 3. Communication | .41  (.29) | –.92  (.58) | –.26^b^ | .25^b^ | -- |

^a^ *P* < .01;
^b^ *P* < .05.

**Table S3.** Conditional effects of the focal predictor at values of the moderator

| Sanctions | Effect | SE | CI | |
| --- | --- | --- | --- | --- |
|  |  |  | Upper | Lower |
| 0 | -.14 | .12 | –.38 | .09 |
| 1 | .67 | .25 | .38 | 1.36 |

**Table S4.** Summary of direct and indirect effects and index of moderated mediation

| Effect | Formula | Value |
| --- | --- | --- |
|  |  |  |
| Direct effect | c' | -.26 |
| Indirect effect (SANC = 0) | (−.41)(−.14) | .06 |
| Indirect effect (SANC = 1) | (−.41)(.87) | -.36 |
| Index of moderated mediation | a*b_3_ (−.41)(1.01) | -.41 |
